# Genomic Characterization of Preclinical Prostate Cancer Cell Line Models

**DOI:** 10.3390/ijms25116111

**Published:** 2024-06-01

**Authors:** Erica L. Beatson, Emily N. Risdon, Giulia C. Napoli, Douglas K. Price, Cindy H. Chau, William D. Figg

**Affiliations:** Genitourinary Malignancies Branch, Center for Cancer Research, National Cancer Institute, National Institutes of Health, Bethesda, MD 20892, USAdkprice@nih.gov (D.K.P.); chauc@nih.gov (C.H.C.)

**Keywords:** prostate cancer, preclinical cell lines, genomics, targeted therapeutics

## Abstract

As we move into the era of precision medicine, the growing relevance of genetic alterations to prostate cancer (PCa) development and treatment demonstrates the importance of characterizing preclinical models at the genomic level. Our study investigated the genomic characterization of eight PCa cell lines to understand which models are clinically relevant. We designed a custom AmpliSeq DNA gene panel that encompassed key molecular pathways targeting AR signaling, apoptosis, DNA damage repair, and PI3K/AKT/PTEN, in addition to tumor suppressor genes. We examined the relationship between cell line genomic alterations and therapeutic response. In addition, using DepMap’s Celligner tool, we identified which preclinical models are most representative of specific prostate cancer patient populations on cBioPortal. These data will help investigators understand the genetic differences in preclinical models of PCa and determine which ones are relevant for use in their translational research.

## 1. Introduction

The development of therapeutic resistance is inevitable for prostate cancer (PCa) patients who progress beyond primary disease, demonstrating an unmet clinical need [1,2]. Sensitivity to standard care is short-lived, and patients develop resistance, resulting in the more aggressive phenotype known as castration-resistant prostate cancer (CRPC) [3,4,5]. Investigating the alterations that occur during disease progression from castration-sensitive to castration-resistant disease could help identify therapeutically relevant targets for this population. As we move into the era of precision medicine, the treatment armamentarium for PCa has expanded with the introduction of targeted compounds, and the number of clinical trials investigating targeted therapies in molecularly stratified patient populations continues to grow.

With the recent approval of PARP inhibitors for DNA damage repair (DDR) biomarker positive CRPC, the use of preclinical models is becoming even more important to explore mechanisms behind drug resistance and cancer progression. However, establishing cell lines from prostate tissue is difficult to achieve, with fewer than 20 new parental models developed in the past 50 years [6,7]. The growing significance of genetic alterations to PCa incidence and treatment [8] reveals the importance of classifying each model at the genomic level. Recent PCa studies have utilized linked-read whole genome sequencing to characterize several cell lines according to mutation status [9], analyze the expression of genes found in the main hormone pathways across six cell models [10], and evaluate the crosstalk between androgen signaling and DNA repair genes in cell lines [11]. Our study characterized the genomic profiles of eight PCa cell lines representative of various stages of disease progression. We aimed to understand the relationship between detected alterations and therapeutic response, specifically in key PCa signaling pathways, including AR signaling, apoptosis, DNA damage repair, PI3K/AKT/PTEN, and tumor suppressor activity. After reviewing available commercial panels and failing to identify an option that included key genes in each of our pathways of interest, we designed a custom AmpliSeq Illumina DNA targeted gene panel. Our analysis led to the identification of novel, previously unrecognized mutations, as well as the validation of known alterations in commonly used PCa cell lines.

## 2. Results

As patient genetic testing becomes more popular and investigators branch into targeted combination therapies, a compilation of the genetic alterations present in actionable dysregulated pathways within five common PCa cell lines (AR-expressing: LNCaP, VCaP, 22Rv1; AR-null/castration-resistant: PC-3 and Du-145) and three daughter cell lines that are castration-resistant (LNCaP-95, LNCaP-abl, and VCaP-CR) will allow for strategic in vitro investigation of the relationship between genetic alterations, targeted therapies, and known PCa mechanisms. Initially, we considered using a commercially available panel for genetic testing, but the available panels did not cover the breadth of the molecular pathways we desired. We therefore designed a custom Illumina DNA Ampliseq panel (Table 1). Our custom panel assessed the mutational status of 20 key genes within known dysregulated pathways in PCa: AR, BCL2, BCL2L1, MCL1, ATM, BARD1, BRCA1, BRCA2, BRIP1, CDK12, CHEK2, NBN, PALB2, RAD51B, AKT1, MAP3K1, PIK3CA, PTEN, MDM2, and TP53 (Table 2). We detected alterations including single nucleotide variants (SNVs), multiple nucleotide variants (MNVs), deletions (DELs), and insertions (INSs) across the coding region of our 20 genes and reported all missense mutations regardless of pathogenic status.

### 2.1. Database Confirmation of Detected Coding Region Alterations

Detected alterations within the coding regions of the 20 sequenced genes in the PC-3, 22RV1, Du-145, LNCaP, and VCaP cell lines were cross-checked with genomic data available publicly via the Broad Cancer Cell Line Encyclopedia (CCLE) on the DepMap portal, the Catalogue of Somatic Mutations in Cancer (COSMIC) database [12], and the Physical Sciences Oncology Network’s (PS-ON) Genomic Characterization of Cell Lines dataset.

#### 2.1.1. PC-3 Cells

**Figure 1:**
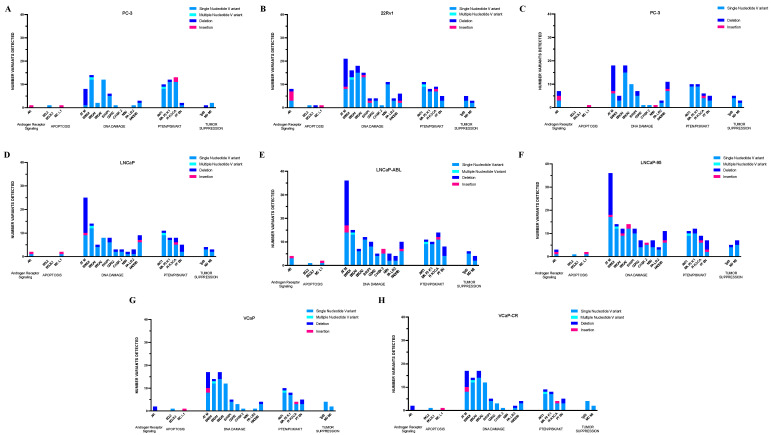
Frequency and type of variants within the coding and non-coding regions of 20 genes commonly dysregulated in PCa, irrespective of clinical and functional significance. Sequencing results for (**A**) PC-3, (**B**) 22Rv1, (**C**) Du-145, (**D**) LNCaP, (**E**) LNCaP-abl, (**F**) LNCaP-95, (**G**) VCaP, and (**H**) VCaP-CR cells.

Within the PC-3 cell line (Figure 1A), our panel detected 8 missense mutations within the coding regions of *BARD1* (3), *MAP3K1* (2), *ATM* (1), *BRCA2* (1), and *BRIP1* (1). One in-frame deletion in *MAP3K1*, one frameshift variant in *TP53*, and one in-frame insertion in *AR* were also detected. All variants, except for the *AR* insertion, were confirmed present in the PS-ON Genomic Characterization dataset. The *TP53* frameshift variant was further confirmed to be present in the CCLE and COSMIC datasets.

#### 2.1.2. 22RV1 Cells

Our panel detected 21 missense mutations within the 22RV1 cell line in the following genes (Figure 1B): *BARD1* (3), *PIK3CA* (1), *MAP3K1* (1), *NBN* (2), *ATM* (2), *BRCA2* (2), *PALB2* (1), *TP53* (2), *BRCA1* (4), *BRIP1* (1), and *AR* (2). All missense mutations, except for P72R in *TP53* and L57Q in *AR*, were confirmed to be present in the PS-ON, CCLE, or COSMIC datasets. Additionally, one frameshift mutation was detected in each *BARD1*, *BRCA2*, and *TP53*. Each of these frameshift mutations was confirmed to be present in the CCLE and COSMIC datasets. The frameshift mutations in *BRCA2* and *TP53* were also confirmed to be present in the PS-ON dataset. Similarly, several in-frame insertions or deletions were also detected within the 22RV1 cell line. One in-frame deletion was detected in the coding region of *MAP3K1* (confirmed in PS-ON), and one in-frame deletion was detected in *AR.* This in-frame deletion in *AR* was not present in any of the three datasets. Finally, we detected four in-frame insertions within *AR*, none of which were present in the three datasets.

#### 2.1.3. Du-145 Cells

Within the Du-145 cell line (Figure 1C), our panel detected 18 missense mutations within the coding regions of the following genes: *MAP3K1* (3), *ATM* (2), *BRCA2* (2), *PALB2* (1), *TP53* (3), *BRCA1* (5), and *BRIP1* (2). One *MAP3K1* missense mutation (N255S) was not confirmed present in any of the three datasets. Of the remaining 17 missense mutations, 2 (P223L in *TP53* and T132N in *BRIP1*) are not present in the PS-ON dataset. The two mentioned missense mutations are present in the CCLE and COSMIC datasets, however. Three other missense mutations were confirmed present in CCLE and COSMIC in addition to PS-ON: S2284L in *BRCA2*, V274F in *TP53*, and E962K in *BRCA1*. We also detected one in-frame deletion in *MAP3K1* that is present in the PS-ON dataset, two in-frame deletions in *AR*, and two in-frame insertions in *AR.* The deletions and insertions detected in the *AR* gene were not present in any of the three datasets.

#### 2.1.4. LNCaP Parental and Derivative Cell Lines

Our panel identified several differences between the three members of the LNCaP family, with the parental line having the fewest alterations. Within the LNCaP cell line (Figure 1D), 16 missense mutations were detected in the following genes: *BARD1* (3), *MAP3K1* (2), *NBN* (1), *ATM* (2), *BRCA2* (2), *RAD51B* (1), *TP53* (1), *CDK12* (1), *BRIP1* (1), *CHEK2* (1), and *AR* (1). All but three of the detected missense mutations (D95N in *NBN*, P72R in *TP53*, and T387N in *CHEK2*) were confirmed in the PS-ON dataset.

In addition to the 16 missense mutations detected, our panel identified 1 in-frame deletion in *MAP3K1*, 1 frameshift deletion in *PTEN*, and 1 in-frame insertion in *AR.* The alterations in *MAP3K1* and *PTEN* were confirmed present in PS-ON. The frameshift mutation in *PTEN* (K6Rfs*4) was also confirmed in the CCLE and COSMIC datasets. We initially sequenced DNA extracted from banked LNCaP cells that had been passaged six times within our lab. However, we have previously shown that the LNCaP cell line easily acquires genetic alterations during culturing [13], prompting us to sequence DNA extracted from an original ATCC vial of LNCaP cells. After completing a second round of sequencing, we found one additional alteration (F55L in *AKT1*) that we did not initially detect.

For the LNCaP-abl cell line (Figure 1E), our panel identified 24 missense mutations within the coding regions of the following genes: *BARD1* (3), *PIK3CA* (1), *MAP3K1* (2), *NBN* (2), *ATM* (2), *BRCA2* (2), *RAD51B* (1), *AKT1* (1), *TP53* (2), *CDK12* (2), *BRIP1* (2), *BLC2* (1), *CHEK2* (2), and *AR* (1). In addition, we detected one in-frame deletion in *MAP3K1*, one in-frame insertion in *AR*, and one stop-gain in *P1K3CA.* Within the LNCaP-95 cell line (Figure 1F), our panel found 25 missense mutations within the coding regions of the following genes: *BARD1* (2), *PIK3CA* (1), *MAP3K1* (2), *NBN* (1), *ATM* (3), *BRCA2* (3), *RAD51B* (1), *AKT1* (1), PALB2 (1), TP53 (1), *CDK12* (1), BRCA1 (2), BRIP1 (2), *BLC2* (1), *CHEK2* (2), and *AR* (1). In addition, we identified one in-frame deletion in *MAP3K1*, one in-frame deletion in *AR*, one in-frame insertion in *AR*, and one stop-gained in *ATM*. We also found one frameshift variant in each *RAD51B*, *CDK12*, and *BRCA1*. Genomic information of both cell lines is not available on the public databases (i.e., CCLE or COSMIC).

#### 2.1.5. VCaP Parental and Derivative Cell Line

For VCaP cells (Figure 1G), our panel detected 17 missense mutations within the coding regions of the following genes: *BARD1* (3), *MAP3K1* (2), *ATM* (3), *BRCA2* (1), *AKT1* (1), *TP53* (2), *BRCA1* (4), and *BRIP1* (1). All but two mutations (F55L in *AKT1* and P72R in *TP53*) were confirmed present in the PS-ON dataset. R248W in *TP53* was further confirmed in the CCLE and COSMIC datasets. In addition to the 17 missense mutations detected in the VCaP Cell line, we detected 1 in-frame deletion in *MAP3K* that was confirmed present in the PS-ON dataset and 2 in-frame deletions in *AR* that were not present in any of the three datasets.

Our panel observed similar alterations within queried genes in the VCaP and VCaP-CR cell lines (Figure 1H), with only one difference observed in *AKT1.* The point mutation resulting in the protein change F55L in *AKT1* was observed in the VCaP cell line but not the VCaP-CR line. While still above standard sequencing cutoffs, the quality score for F55L in the VCaP cell line was 98, and the variant allele frequency was reported to be around 5%. More tests are needed to determine the validity and effects of this reported alteration.

### 2.2. Clinically Actionable Alterations in Selected Pathways

To assess the clinical relevance of alterations detected with our panel, we consulted ClinVar and OncoKB when available. In the cases where no established entries exist for certain detected alterations, we used four in silico prediction engines: SIFT [14], PolyPhen-2 [15,16], Mutation Assesor [17,18], and FATHMM [19,20] to predict alteration-driven protein functional changes using sequence homology and the physical interactions between amino acids.

#### 2.2.1. AR Pathway

**Figure 2:**
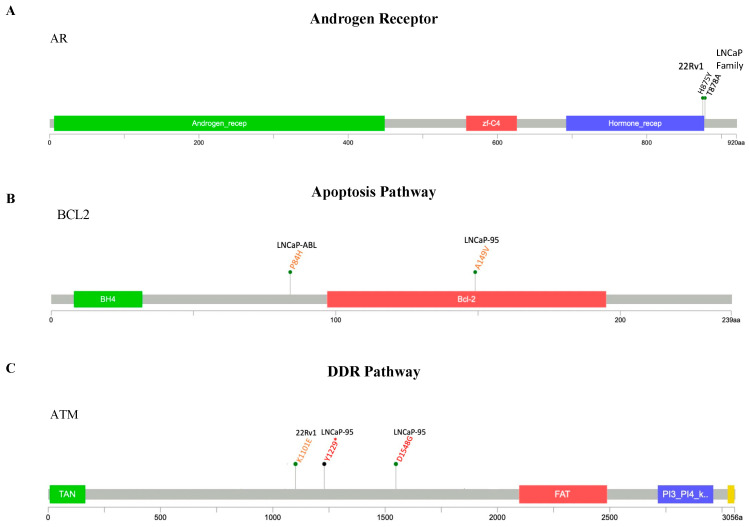

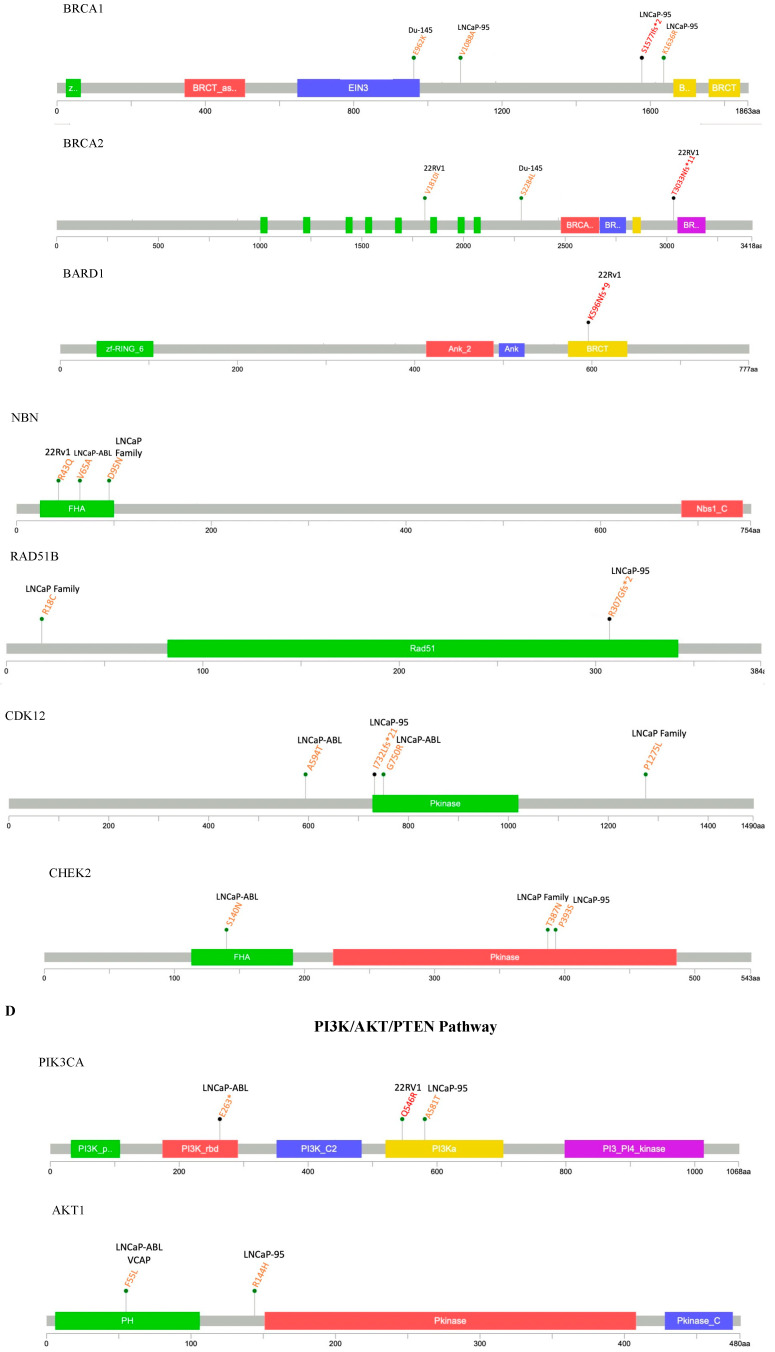

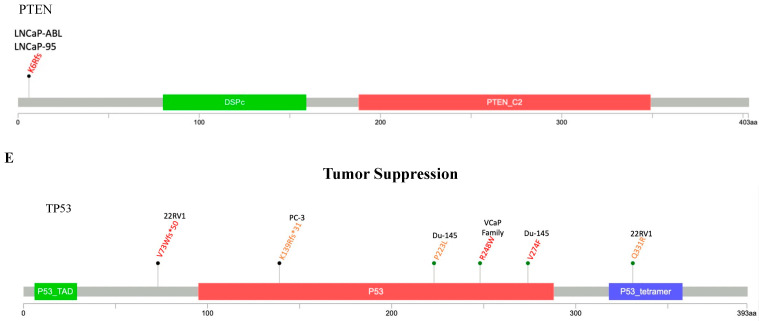
Clinically actionable alterations in key dysregulated pathways in PCa cell line models. Lollipop plots describe gene variants within the (**A**) AR, (**B**) apoptosis, (**C**) DDR, (**D**) PI3K/AKT/PTEN, and (**E**) tumor suppression pathways. Red font of variant description indicates the variant is confirmed to be pathogenic by ClinVar and orange indicates conflicting results from ClinVar and prediction engines. Lollipop plots mapping coding region mutations were produced using cBioPortal’s MutationMapper.

Reactivation of the AR following ADT is a hallmark event of PCa progression to CRPC. There are many mechanisms by which reactivation occurs, including AR amplification, over-expression, the presence of constitutively active variants, and mutation [21,22,23]. The LNCaP family (LNCaP, LNCaP-abl, and LNCaP-95) and 22Rv1 cell lines each harbor a well-characterized, gain-of-function missense mutation within the coding region of *AR* (T878A and H875Y, respectively), which allows the AR to be activated by progesterone, estrogen, flutamide, bicalutamide, enzalutamide, and apalutamide [24,25,26] (Figure 2A). The H875Y alteration allows the AR to also be activated by glucocorticoids and adrenal androgens [23,27,28,29]. While we did not find any definitively pathogenic *AR* alterations within the PC-3, Du-145, or VCaP family cell lines, several frameshift mutations with unknown consequences were identified.

#### 2.2.2. Apoptosis Pathway

Our panel covered three genes involved in regulating apoptosis: *BCL2*, *BCL2L1* (BCL-XL), and *MCL1*. While we did not detect any alterations within the coding regions of *BCL2L1* or *MCL1* in any of the eight cell lines examined, our panel detected two missense mutations within *BCL2* in the LNCaP-abl and LNCaP-95 cell lines (P84H and A149V, respectively) that were predicted to be pathogenic or possibly pathogenic by the in silico prediction engines MutationAssesor, SIFT, and PolyPhen-2 (Figure 2B). Neither of these alterations are well characterized, however, and the consequences of the resulting protein changes are currently unknown.

#### 2.2.3. DDR Pathway

We designed our panel to sequence ten genes with key roles in DDR: *ATM*, *BARD1*, *BRCA1*, *BRCA2*, *BRIP1*, *CDK12*, *CHEK2*, *NBN*, *PALB2*, and *RAD51B* (Figure 2C). In the 22Rv1 cell line, we identified alterations with suspected clinical implications in several genes coding for key proteins within the major DDR pathways: *ATM*, *BRCA2*, *BARD1*, *NBN*, *PALB2*, and *BRIP1.* Within the *BRCA2* gene, our panel detected an SNP (V1810I) and a truncating mutation at residue 3033 (T3033NFs*11) that likely results in the loss-of-function of BRCA2. In the *BARD1* gene, a truncating mutation within the BRCA1 C terminus (K596Nfs*11) was detected, within the *NBN* gene, a missense mutation at residue 43 (R43Q) within the fork-head associated (FHA) domain was detected, and mutations in the *PALB2* (V1123M) and *BRIP1* (R173C) genes were detected in 22RV1 cells. Within the Du-145 cell line, we detected a potentially deleterious *BRCA2* missense mutation at residue 2248 (S2284L) and an alteration in *BRCA1* (E962K).

The LNCaP family of cell lines harbor several shared missense mutations with pathogenic potential within key DDR genes: *NBN* (D95N), *RAD51B* (R18C), *CDK12* (P1275L), and *CHEK2* (T387N). DDR alterations with pathogenic potential exclusive to the LNCaP-95 cell line include a stop-gain alteration in *ATM* (Y1229*), missense mutations in *ATM* (D1548G), *PALB2* (Q1114R), *BRCA1* (V1088A and K1636R), *BRIP1* (G290S), and CHEK2 (P393S), and frameshift alterations in *RAD51B* (R307Gfs*2), *CDK12* (I732Lfs*21), and *BRCA1* (S1577Ifs*2). Potentially pathogenic alterations within DDR pathway genes exclusive to the LNCaP-abl cell line include missense mutations in *NBN* (V65A), *CDK12* (G750R and A594T), *BRIP1* (Q322R), and *CHEK2* (S140N).

The VCaP family of cell lines harbor several missense mutations in DDR genes, none of which are expected to affect protein function.

#### 2.2.4. PTEN/PI3K/AKT Pathway

Our panel was designed to investigate the alterations in *PIK3CA*, *AKT1*, and *PTEN* (Figure 2D). Of the eight PCa cell lines sequenced, *PIK3CA* is notably altered in 22Rv1 (Q546R), LNCaP-95 (A581T), and LNCaP-abl (E263*) cells. Three PCa cell lines (VCaP, LNCaP-abl, and LNCaP-95) have a potential gain-of-function mutation in *AKT1* (F55L). Interestingly, DNA was initially isolated from LNCaP cells that had been passaged six times, and the sequencing results did not show the alteration. A second round of sequencing with DNA derived from younger cells at low passage resulted in the detection of the alteration F55L in *AKT1*, indicating that the activating alteration is lost during subculture. This suggests that cell lines derived from the parental line may serve as a better model for studying the AKT pathway, as the activating mutation remains present in the LNCaP-abl derivative. Finally, the LNCaP, LNCaP-abl, and LNCaP-95 cell lines exhibit a frameshift mutation (K6Rfs*4).

#### 2.2.5. Tumor Suppression Pathway

The VCaP, VCaP-CR, Du-145, 22RV1, and PC-3 cell lines each contain alterations in *TP53* (Figure 2E). The VCaP and VCaP-CR cell lines contain the missense mutation R248W. The Du-145 cell line contains two likely loss-of-function amino acid changes, V274F and P223L. The 22RV1 cell line contains the frameshift deletion V73Wfs*50 and a missense mutation Q331R, which are expected to result in p53’s ability to function as a tumor suppressor. Finally, the PC3 cell line harbors a frameshift deletion K139Rfs*31 that is expected to be deleterious. None of the eight cell lines screened contained confirmed or suspected function-altering aberrations in *MDM2*.

### 2.3. Therapeutic Targeting of Cell Line-Specific Markers

Following sequencing and analysis of variants, we considered alterations present in each pathway per cell line for results warranting further investigation. Due to the varying frequency of alterations between the parent and daughter cell lines within dysregulated PCa pathways, we treated the LNCaP family cell lines with the PARP inhibitor olaparib, PI3K inhibitors alpelisib and GDC-0077, and AKT inhibitors ipatasertib, capivasertib, and A-674563.

**Figure 3:**
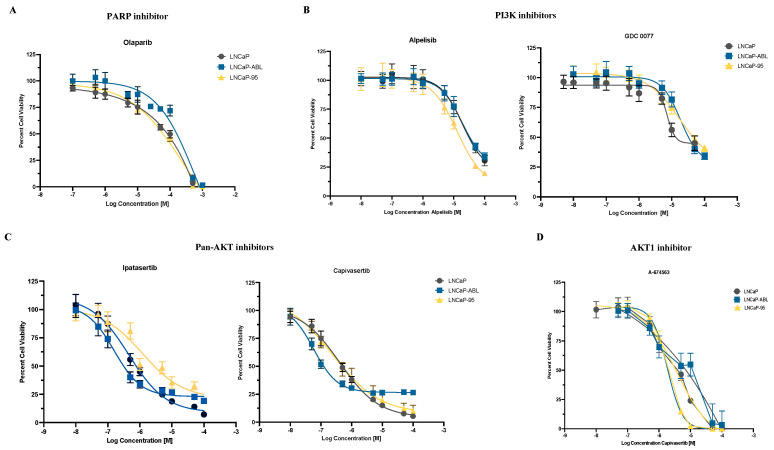
The LNCaP family has varying sensitivity to compounds targeting PCa clinically actionable pathways. Parental LNCaP, LNCaP-abl, and LNCaP-95 cells were treated with a PARP inhibitor (**A**), PI3K inhibitors (**B**), pan-AKT inhibitors (**C**), or an *AKT1* inhibitor (**D**). Cell viability was determined after 48 h of treatment by CellTiter-Glo luminescent assay. Data provided represent mean ± standard deviation from three individual experiments.

Due to the high volume of alterations detected within essential DDR genes in the LNCaP family cell lines, we tested LNCaP parental, LNCaP-95, and LNCaP-abl cell lines with a range of concentrations of the PARP inhibitor olaparib (Figure 3A). The varied DDR alteration landscapes of the three LNCaP family cell lines contributed marginally to the slight shifts in the IC_50_ curves observed (IC_50_> 10 µM).

Each of the three LNCaP family cell lines exhibit a varying *PIK3CA/AKT1* alteration profile. The LNCaP parental does not show any potential or confirmed deleterious alterations, while the LNCaP-95 and LNCaP-abl each contain alterations with potentially pathogenic consequences in *PIK3CA* and *AKT1.* Additionally, the LNCaP family cell lines do not express PTEN protein [30]. For these reasons, we treated each LNCaP family cell line with the PI3K inhibitors alpelisib and GDC-0077, the pan-AKT inhibitors ipatasertib and capivasertib, and the AKT1-specific inhibitor A-674563.

The LNCaP-95 and LNCaP-abl cell lines both exhibit alterations within the coding region of *PIK3CA*. LNCaP-95 cells harbor the alteration A581T within the helical region of *PIK3CA* that could potentially result in a gain-of-function of PI3K, while LNCaP-abl cells have a stop-gain alteration E263*, the effects of which are unknown. We treated all three LNCaP family cell lines with the PI3K inhibitors alpelisib and GDC-0077 (Figure 3B), which are known to elicit increased responses in *PIK3CA*-altered settings [31,32]. Treatment with alpelisib resulted in similar responses in the LNCaP parental and LNCaP-abl cell lines with IC50s of ~20 µM. Interestingly, LNCaP-95 cells elicited a slightly better sensitivity with an IC_50_ of ~13 µM. Treatment with GDC-0077 demonstrated a similar response in LNCaP-abl and LNCaP-95 cells (IC50s of ~20 µM) while LNCaP parental cells responded with an IC50 of ~7 µM. More tests would need to be run to determine whether the changes in sensitivity observed in the LNCaP family result from the A581T alteration in *PIK3CA*.

Our panel detected one variant each within the coding region of *AKT1* in the LNCaP-95 and LNCaP-abl cell lines, R144H and F55L, respectively. The effects of these alterations are unknown. We sought to identify whether either of the alterations resulted in sensitivity to AKT inhibitors, potentially due to a pathogenic gain-of-function event within its associated cell line. The LNCaP family cell lines displayed sensitivity to treatment with the pan-AKT inhibitors ipatasertib and capivasertib, with the LNCaP-abl cell line appearing to be more sensitive than the parental line and LNCaP-95 (Figure 3C). Treatment with an *AKT1*-specific compound, A-674563, revealed LNCaP-95 cells to be more sensitive to *AKT1* inhibition than the LNCaP-abl and LNCaP parental cell lines, with an IC-50 of 1.6 µM compared to 5.3 µM and 3.4 µM, respectively (Figure 3D). Whether or not the F55L and R144H alterations contribute to the responses observed in these screens deserves further investigation, along with considering potential contributions from the uninterrogated subunits of both AKT and PI3K.

### 2.4. The Cancer Dependency Map Celligner Tool

**Figure 4:**
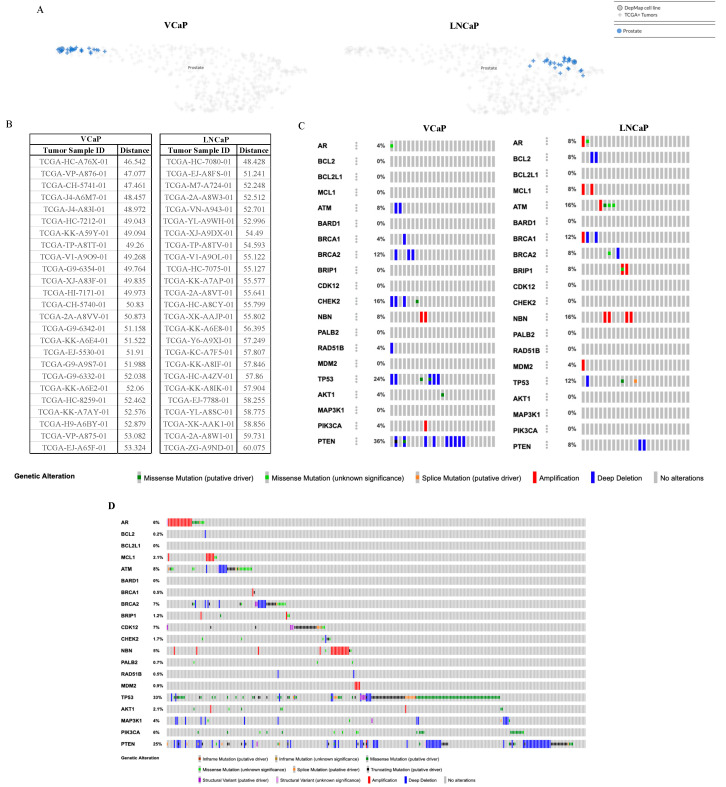
Alteration profile comparison between preclinical models and clinical samples from PCa patients. (**A**,**B**) Celligner alignment of cancer cell lines and tumor samples. UMAP visualization showing the genome-wide gene expression-based alignment of VCaP and LNCaP cell lines compared to patient tumor gene expression data. Representation was created via the DepMap Celligner tool. Blue circles represent cell lines and blue plus signs represent closely related patients. (**C**) cBioPortal OncoPrint of queried genes using whole-exome sequencing of TCGA primary cancer patients identified as closely related to VCaP and LNCaP cells and of (**D**) mCSPC MSK patient population analyzed by Stopsack et al. [34].

We next determined how representative the cancer cell lines are to the prostate cancer patient population. The Cancer Dependency Map (Depmap) offers the computational tool Celligner, which compares preclinical models to their intended patient populations [33]. The analysis measures differences in expression profiles between cell lines and available patient samples from the TCGA cohort to produce a Pearson correlation distance value, which characterizes the similarities between the model and the population. DepMap has data available for our cell lines of interest, of which we found the closest 25 neighbors for both VCaP and LNCaP are PCa patients from the TCGA cohort of primary prostate adenocarcinoma patients (Figure 4A). The 25 corresponding patients for VCaP have a tighter clustered distance range compared to LNCaP, suggesting that it may serve as a more representative preclinical model (Figure 4B); however, both cell lines exhibit more mutations than are represented by their corresponding 25 patients. Oncoprints generated by cBioPortal demonstrate that VCAP deviates from the patient profiles with additional alterations to *BCL2*, *MCL1*, *BARD1*, *BRIP1*, *CDK12*, *PALB2*, *MDM2*, *AKT1*, and *MAP3K1* and no amplification at *NBM*. LNCaP similarly had other mutations to *BARD1*, *CDK12*, *CHEK2*, *PALB2*, *RAD51B*, *AKT1*, *MAP3K1*, and *PIK3CA*, but did not share the same mutation in *BCL2* with its mapped patient cohort (Figure 4C). Both VCaP and LNCaP cell lines are androgen-dependent and originate from metastatic sites. Therefore, both models should have mutation profiles that match metastatic, castration-sensitive PCa (mCSPC). Compared to mCSPC genomic data from the study conducted by Stopsack et al. (designated as the Memorial Sloan Kettering (MSK) patient cohort, *n* = 424 samples) available on cBioPortal [34], we observed that VCaP cells were more closely aligned with the mutation rates of mCSPC patients than LNCaP cells (Figure 4D). Specifically, mutation rates for *BCL2*, *MCL1*, *ATM*, *BRCA1*, *BRIP1*, *NBN*, *MDM2*, *TP53*, *PIK3CA*, and *PTEN* from VCaP cells more closely resembled the profile of MSK patients. This further suggests that VCaP may better represent this subsect of patients.

## 3. Discussion

Our study characterized the array of preclinical PCa cell lines using a custom AmpliSeq DNA gene panel. We aimed to identify the alteration status in key genes involved in AR signaling, apoptosis, DNA damage repair, and PI3K/AKT/PTEN, in addition to tumor-suppressing genes. Our results contributed to prior studies’ efforts to characterize the most common human PCa cell lines [9,10,11]. We selected and tested targeted compounds using our results to identify whether mutation status predicted therapeutic sensitivity. Finally, we determined which cell lines best represent PCa populations using DepMap celligner and publicly available genomic data visualized by cBioPortal.

Cellular response to DNA damage usually occurs through some variation in one or more of the five canonical DDR pathways: homologous recombination (HR), non-homologous end joining (NHEJ), base excision repair, nucleotide excision repair, or mismatch repair. Pathway combination and use is determined by the timing, source, type, and extent of damage [35,36]. Mutations in genes coding for protein members of DDR mechanisms that result in impaired function can be targeted and taken advantage of to create advantageous lethal events in tumor cells, a concept known as synthetic lethality [37,38]. This can be accomplished by using PARP inhibitors to treat patients who have germline or somatic alterations in homologous recombination genes. While the potential benefit of PARP inhibitor treatment in BRCA-deficient patients is well documented, there is compelling evidence that patients with alterations in other HR and DDR genes might also benefit from PARP inhibitor treatment [39].

Within the *BRCA2* gene, our panel detected V1810I and a truncating mutation at T3033NFs*11 that likely results in the loss-of-function of BRCA2. T3033Nfs*11 has been detected in Fanconi anemia patients, as well as in breast, ovarian, and pancreatic cancer patient populations [40,41,42,43]. The effects of V1810I are currently unknown, but MutationAssesor predicts this alteration to have a medium likelihood of pathogenicity. In the *BARD1* gene, a truncating mutation within the BRCA1 C terminus (K596Nfs*11) was detected that may impair BARD1 activity and interactions with BRCA1 [44,45]. Within the *NBN* gene, a missense mutation at residue 43 (R43Q) within the fork-head associated (FHA) domain was detected. SIFT, PolyPhen-2, and Mutation Assessor each flagged R43Q to have a high likelihood of being deleterious. NBN’s FHA domain is essential for the protein’s interaction with DNA damage and recruitment of other repair factors [46]. Finally, mutations in the *PALB2* (V1123M) and *BRIP1* (R173C) genes were detected in 22RV1 cells and are predicted to be deleterious by the in silico prediction engines. Within the Du-145 cell line, we detected a potentially deleterious *BRCA2* missense mutation at residue 2248 (S2284L). SIFT predicts this missense mutation to be deleterious, but other predictions of this change’s effects are ambiguous. The Du-145 cell line also harbors an alteration in *BRCA1* (E962K) that has uncertain consequences. Two prediction engines, PolyPhen-2 and Mutation Assessor, predict E962K to possibly have pathogenic effects.

The PI3K/AKT signaling pathway is implicated in various cellular processes, including cell survival, growth, and proliferation [47]. PTEN acts as a mechanistic break of the PI3K-AKT signaling axis through its dephosphorylation of PIP3 to PIP2. Taylor et al. determined the PI3K pathway is altered in 42% of primary PCa cases and 100% of metastatic PCa cases [48]. PI3K’s p110α catalytic subunit is encoded by the *PIK3CA* gene that contains alterations known to impact the PI3K/AKT signaling axis in various cancer types [49,50]. Of the eight PCa cell lines sequenced, *PIK3CA* is altered in 22Rv1 (Q546R), LNCaP-95 (A581T), and LNCaP-abl (E263*) cells. Both Q546R and A581T are missense mutations in the helical domain of *PIK3CA*. Alterations in *PIK3CA*’s helical domain are associated with a gain-of-function of PI3K. This effect is well characterized for Q546R, an alteration that results in higher cell proliferation, migration, and survival [51], but less so for A581T. The in silico prediction engines SIFT and FATHMM predict A581T to have deleterious effects. More tests are necessary to determine whether A581T affects PI3K’s function. Downstream from PI3K, the AKT kinase comprises three closely related serine/threonine protein kinases: AKT1, AKT2, and AKT3 [52,53,54]. Of the eight PCa cell lines sequenced, three have a potential gain-of-function mutation in *AKT1* (F55L). This alteration occurs in the pleckstrin homology (PH) domain and was initially found in LNCaP-abl, LNCaP, and VCaP cells. Alterations in AKT1’s PH domain interrupt the PH–kinase domain (KD) interaction that maintains AKT1 in an inactive state in basal conditions [55,56,57,58]. While the relationship between F55L and AKT activation has not been explicitly described previously, Parikh et al. showed that a similar mutation, F55Y, disrupts the PH-KD interaction and likely results in AKT activation [58]. Whether PI3K or AKT mutations predict drug sensitivity remains to be further tested clinically.

PTEN acts as a regulator of PI3K’s activation of AKT through its conversion of phosphatidylinositol 3,4,5-trisphosphate (PI(3,4,5)P3 or PIP3) into phosphatidylinositol 4,5-bisphosphate (PI(4,5)P2 or PIP2) [59]. *PTEN* loss is associated with increased PI3K-AKT signaling and observed in around 40% of metastatic PCa sites examined in several studies [48,60,61,62]. The LNCaP, LNCaP-abl, and LNCaP-95 cell lines exhibit a frameshift mutation (K6Rfs*4) that is expected to result in PTEN loss-of-function.

The tumor suppressor *TP53* is altered in more than 50 percent of human cancers, including PCa [63,64,65]. One mechanism of deactivation of p53 is through a feedback loop with MDM2, an inhibitor of the protein that becomes overexpressed [63]. When functional, p53 is best characterized to be involved in cell cycle arrest, senescence, and apoptosis [66]. The protein blocks progression beyond the G1 phase after the detection of DNA damage by inducing the expression of cyclin-dependent kinase inhibitor 1A (*CDK1A*). Cell cycle arrest allows for either damage repair or apoptosis through downstream interactions with the pro-apoptotic Bcl-2 family [66]. The VCaP and VCaP-CR lines each contain alterations in *TP53* that are known to be damaging. The cell lines contain the missense mutation R248W, a well-characterized alteration that results in the loss of p53’s ability to function as a tumor suppressor [67]. Song et. al. validated that the p53 alteration has oncogenic effects by introducing R248W mutations into the humanized mice with a p53 knock-in allele [68]. Specifically, they found that p53 has downstream effects on the binding of the MRN complex to double-stranded breaks, which then prevent *ATM* from activating the G2-M checkpoint to check for DNA damage [68].

As proven by the success of PARP inhibitors in PCa, mutation status can indicate sensitivity to specific targeted therapies. The necessity of biomarker status translates back to the preclinical realm, where cell line models are used to evaluate the efficacy of targeted compounds and, therefore, must accurately represent the intended patient population. Our results identify the alteration status of 20 genes commonly implicated in PCa development, many of which are also the target of up-and-coming therapies being tested for anticancer effects. Considering the mutation profile of LNCaP-95 cells, we evaluated the potency of PI3K and AKT inhibitors within the LNCaP family. We found that alpelisib and A-674563 were the most sensitive in LNCaP-95 cells compared to LNCaP and LNCaP-abls. More studies would need to be conducted to identify whether alterations to *PIK3CA* or *AKT1* have implications for drug sensitivity or resistance in the LNCaP family. Future work should likewise consider the alteration status while evaluating drug efficacy in PCa models.

Preclinical models provide the foundation for studying cancer development and identifying viable treatment options. To learn about therapeutic efficacy and mechanisms of resistance, we need to understand the genomic and proteomic landscape of our cell lines before translating this knowledge to the clinic. Our study validated many of the alterations acknowledged by CCLE, COSMIC, and PS-ON and found previously unidentified mutations across our 20 genes of interest. Using DepMap’s celligner tool, we isolated 25 patients from the TCGA cohort who most closely resemble the genomic profile of both VCaP and LNCaP cells. We were also able to compare the alteration profile of the two cell lines to the MSK mCSPC patient cohort on cBioPortal, concluding that VCaP cells may serve as a close representative model for CSPC patients for preclinical studies. Our results will support future researchers as they choose PCa models to best represent their intended patient population.

Ultimately, this study characterized the alteration status of 20 genes found within known dysregulated molecular pathways in PCa across eight preclinical cell lines. Our results confirm the validity of these models in representing the population of PCa patients accurately, which is necessary in the current era of precision medicine to identify preclinical models that are clinically relevant for translational research.

## 4. Materials and Methods

### 4.1. Cell Culture

PCa cell lines PC-3 (RRID: CVCL_0035), Du-145 (RRID: CVCL_0105), LNCaP (RRID: CVCL_1379), 22Rv1 (RRID: CVCL_1045), and VCaP (RRID: CVCL_2235) were purchased from American Type Culture Collection (ATCC) (Manassas, VA, USA). The LNCaP-95 (RRID: CVCL_ZC87) and VCaP-CR (RRID: CVCL_A0BX) cell lines were kindly gifted by Dr. Jun Luo (Johns Hopkins Biobank). The LNCaP-abl (RRID: CVCL_4793) cell line was kindly provided by Dr. Zoran Culig. Unless stated otherwise, cell culture reagents were purchased from Gibco/ThermoFisher Scientific (Waltham, MA, USA). The LNCaP and 22Rv1 cell lines were maintained in phenol red-free 1640 RPMI medium supplemented with 10% fetal bovine serum (FBS, Bio-techne, Minneapolis, MN, USA), 100 units/mL penicillin, 100 units/mL streptomycin (1% P/S). PC-3 cells were maintained in F-12K media supplemented with 10% FBS and 1% P/S. The LNCaP-95 and LNCaP-abl cell lines were maintained in phenol red-free 1640 RPMI medium supplemented with 10% charcoal dextrin-stripped fetal bovine serum (CDS-FBS, Bio-techne), 1% P/S. The Du-145 and VCaP cell lines were maintained in DMEM media supplemented with 10% FBS, 1% P/S. The VCaP-CR cell line was maintained in phenol red-free 1640 RPMI media supplemented with 10% CDS-FBS and 2% B-27 neuronal supplement and grown on Cell+ cell culture flasks (Sarstedt, Nümbrecht, Germany). Cells were maintained at 37 °C in an atmosphere containing 5% CO_2_ and 95% humidity. Cell line authenticity and mycoplasma contamination were routinely performed by STR genotyping and PCR-based assays (ATCC).

### 4.2. DNA Extractions

Cells from each cell line (thawed from an original ATCC vial) were detached and resuspended in associated complete media, counted using the Countess II Automated Cell Counter system and associated reagents (ThermoFisher Scientific), and resuspended to 1–2 million cells/mL. DNA was extracted using the Gentra Puregene Cell Kit (Qiagen, Germantown, MD, USA) according to the manufacturer’s instructions. DNA was quantified using NanoDrop 1000 (ThermoFisher), diluted to 50–100 ng/µL in Low TE (Illumina, Inc., San Diego, CA, USA), and re-quantified using the Qubit dsDNA High Sensitivity assay kit (Invitrogen, Waltham, MA, USA) according to the manufacturer’s instructions. DNA was then diluted to a final concentration of 4 ng/µL in Low TE (Illumina, Inc.).

### 4.3. Custom Illumina AmpliSeq Panel Construction

A custom AmpliSeq DNA Gene panel was designed using Illumina, Inc.’s DesignStudio. The finalized panel covers 99.89% of 20 genes of interest: *AR*, *BCL2*, *BCL2L1*, *MCL1*, *ATM*, *BARD1*, *BRCA1*, *BRCA2*, *BRIP1*, *CDK12*, *CHEK2*, *NBN*, *PALB2*, *RAD51B*, *AKT1*, *MAP3K1*, *PIK3CA*, *PTEN*, *MDM2*, and *TP53*. The panel contains 455 amplicons in two pools (228/227) with an average amplicon length of 357 base pairs. Amplicon targets and probes can be found in Table S1.

### 4.4. Library Preparation and Targeted Next-Generation Sequencing

All reagents used for library preparation and targeted NGS were purchased from Illumina, Inc. (USA) unless otherwise stated. A custom protocol was generated using Illumina’s Custom Protocol Selector tool. A mass of 20 ng of DNA total (10 ng/pool) was input for each sample. Targets were amplified using the AmpliSeq Library PLUS (24 reactions) for the Illumina kit per the manufacturer’s instructions. Indexes were ligated using the AmpliSeq UD Indexes for the Illumina kit per the manufacturer’s instructions. The libraries were cleaned and equalized using the AmpliSeq Library Equalizer for Illumina kit and Agencourt AMPure XP Beads (Beckman Coulter, Brea, CA, USA) according to the manufacturer’s instructions. Libraries were confirmed and analyzed for quality control using the 2100 Bioanalyzer instrument (Agilent, Santa Clara, CA, USA) and Agilent High Sensitivity DNA Kit (Agilent) per the manufacturer’s instructions. Following confirmation and quality control, 10 µL of each library was pooled, denatured, and diluted according to Protocol D of the MiSeq System Denature and Dilute Libraries Guide (Illumina, Inc.). The pooled library suspension was then supplemented with 1% denatured 20 pM PhiX control for a final volume of 600 µL. Pooled sample libraries were loaded into an Illumina MiSeq V3 reagent cartridge according to the manufacturer’s instructions. Paired-end sequencing of pooled libraries was performed using MiSeq V3 reagents (600-cycle) with a mean amplicon coverage of 9949.4 per sample.

### 4.5. Variant Identification and Analysis

Following sequencing, on-instrument analysis was performed via Local Run Manager. Reads were demultiplexed according to the specified index per sample and stitched to form a single read with a minimum of 10 overlapping base pairs between read one and read two. Clusters were aligned using BWA Aligner (Illumina, Inc.) against amplicon probe sequences listed within the sequencing manifest using the banded Smith–Waterman algorithm. Alignments with more than three indels within the probe sequence were excluded from alignment results. Variant calling was performed using the Pisces Variant Caller (Illumina, Inc., USA). Variants with a quality score below Q30 were excluded. Detected alterations were cross-checked with genomic data available publicly via the Broad Cancer Cell Line Encyclopedia (CCLE), the Catalogue of Somatic Mutations in Cancer (COSMIC) database [12], and the Physical Sciences Oncology Network’s (PS-ON) Genomic Characterization of Cell Lines dataset. Functional consequences of previously annotated variants were investigated using ClinVar, COSMIC, and OncoKB public databases [12,69,70,71]. The prediction engines PolyPhen-2, Mutation Assessor, and SIFT were used to predict functional changes for uncharacterized variants. Lollipop plots mapping coding region mutations were produced using cBioPortal’s MutationMapper.

### 4.6. Compounds

Unless otherwise stated, all agents were purchased from SelleckChem (Houston, TX, USA). LNCaP, LNCaP-abl, and LNCaP-95 cell lines were treated with PARP inhibitor olaparib, AKT inhibitors capivasertib, ipatasertib, and A-674563, and PI3K inhibitors alpelisib and GDC-0077 (MedChemExpress, Monmouth Junction, NJ, USA). Upon receipt, agents were dissolved in DMSO to a stock concentration of 10 mM, sonicated for 15–20 min, and aliquoted and stored at −80 °C.

### 4.7. CellTiter-Glo Luminescent Cell Viability Assay

Cells were seeded (15,000 cells/well for LNCaP-abl and LNCaP-95; 10,000 cells/well for LNCaP) in non-coated or CellBind black-walled, clear-bottom 96-well plates (Corning, Corning, NY, USA) and left to incubate for 24 h at 37 °C in 5% CO_2_. Media was replaced with media containing vehicle (0.5% DMSO) or varying concentrations of test compounds for 48 h of incubation. Cell viability was assessed using CellTiter-Glo (Promega, Madison, WI, USA) according to the manufacturer’s instructions and luminescence was measured on SpectraMax ID3 plate reader (Molecular Devices, San Jose, CA, USA). Results were normalized to the highest % (*v*/*v*) of the DMSO vehicle treatment group.

### 4.8. Population Data

To measure the representative nature of PCa preclinical cell lines to their disease counterparts, we utilized The Cancer Dependency Map’s (Depmap) Celligner tool. Celligner compares the expression profiles of cell lines to publicly available patient data from The Cancer Genome Atlas (TCGA) to identify a narrowed list of patients well represented by a chosen cell line [33]. The comparative analysis was quantified through the Pearson correlation distance, with lower distance values representing a higher level of resemblance.

Sequencing data were downloaded from cBioPortal for metastatic mCSPC patients from the MSK patient cohort [34]. Patient mutation profiles were displayed by oncoprints generated on cBioPortal [72,73,74].

## Figures and Tables

**Table 1:** A custom protocol was developed utilizing Illumina’s Custom Protocol Selector tool to assess the mutation status of 20 pre-identified genes important to PCa incidence and progression.

		Figg Lab Panel	BRCAnalysis CDX	FoundationONE CDX	ProstateNext	Invitae PCa Panel	Color Hereditary Prostate	Fulgent
**Androgen Receptor** **Signaling**	AR	x						
**Apoptosis**	BCL2	x						
	BCL2L1	x						
	MCL1	x						
**DNA Damage Repair**	ATM	x		x	x	x	x	x
	BARD1	x		x				x
	BRCA1	x	x	x	x	x	x	x
	BRCA2	x	x	x	x	x	x	x
	BRIP1	x		x		x		x
	CDK12	x		x				
	CHEK2	x			x	x	x	x
	NBN	x			x	x		x
	PALB2	x		x	x	x		x
	RAD51B	x		x				
**PTEN/PI3K/AKT** **pathway**	AKT1	x						x
	MAP3K1	x						
	PIK3CA	x						x
	PTEN	x						x
**Tumor Suppression**	TP53	x			x	x	x	x
	MDM2	x						

**Table 2:** Sequencing results for commercially available cell lines PC-3, 22Rv1, Du-145, LNCaP, and VCaP cells, in addition to the derivative cell lines LNCaP-abl, LNCaP-95, and VCaP-CR. The Figg Lab panel assessed single nucleotide variant status (SNV), multiple nucleotide variant status (MNV), deletions (DELs), and insertions (INSs) across the coding regions of 20 genes.

	AR	ATM	AKT1	BARD1	BCL2	BCL2L1	BRCA1	BRCA2	BRIP1	CDK12	CHEK2	MAP3K1	NBN	MCL1	MDM2	PALB2	PIK3CA	PTEN	RAD51B	TP53
**PC-3**	INS-1	DEL-7	SNV-8	SNV-12	SNV-1		SNV-2	SNV-12	SNV-5	SNV-1		SNV-11		INS-1	SNV-2	SNV-1	SNV-11	DEL-1	SNV-2	DEL-1
		SNV-1	MNV-1	MNV-1					DEL-1			DEL-1					INS-2	SNV-1	DEL-1	
			DEL-1	DEL-1																
**22RV1**	INS-4	DEL-12	SNV-9	SNV-12	SNV-1	DEL-1	SNV-15	SNV-13	SNV-2	SNV-3	SNV-1	SNV-7	SNV-10	INS-1	SNV-2	SNV-3	SNV-7	SNV-3	SNV-2	SNV-3
	SNV-3	SNV-8	MNV-1	MNV-1			DEL-3	DEL-1	DEL-1	DEL-1		DEL-1	DEL-1		DEL-1	DEL-1	DEL-1	DEL-2	DEL-3	DEL-2
	DEL-1	INS-1	DEL-1	DEL-3				INS-1	INS-1								INS-1		INS-1	
**Du-145**	SNV-3	DEL-11	SNV-9	SNV-3			SNV-15	SNV-10	SNV-5	SNV-1	SNV-1	SNV-9	INS-1	INS-1	SNV-2	SNV-2	SNV-4	SNV-3	SNV-7	SNV-4
	DEL-2	SNV-6	DEL-1	DEL-2			DEL-3		DEL-2			DEL-1			DEL-1	DEL-1	DEL-1	DEL-2	DEL-3	DEL-1
	INS-2	INS-1															INS-1		INS-1	
**LNCaP**	SNV-1	DEL-15	SNV-9	SNV-12			SNV-4	SNV-8	SNV-6	SNV-2	SNV-2	SNV-7	SNV-1	INS- 1	SNV-2	SNV-1	SNV-5	SNV-2	SNV-6	SNV-3
	INS-1	SNV-9	MNV-1	MNV- 1			DEL-1		DEL-2	DEL-1	INS-1	DEL-1	DEL-1	SNV-1	DEL-1	DEL-2	DEL-2	DEL-3	INS-1	DEL-1
		INS-1	DEL-1	DEL-1													INS-1		DEL-2	
**LNCaP-ABL**	SNV-3	DEL-19	SNV-9	SNV-13	SNV-1		SNV-6	SNV-11	SNV-8	SNV-4	SNV-5	SNV-9	DEL-3	INS-1	SNV-2	SNV-2	SNV-11	SNV-4	SNV-6	SNV-5
	INS-1	SNV-14	MNV-1	MNV-1			DEL-1	DEL-1	DEL-2	DEL-1	INS-2	DEL-1	SNV-2	SNV-1	DEL-2	DEL-2	DEL-2	DEL-4	INS-1	DEL-1
		INS-3	DEL-1	DEL-1													INS-1		DEL-3	
**LNCaP-95**	SNV-1	DEL-18	SNV-9	SNV-12	SNV-1		SNV-9	SNV-12	SNV-10	SNV-4	SNV-5	SNV-10	SNV-4	INS-1	SNV-5	SNV-3	SNV-6	SNV-2	SNV-6	SNV-4
	INS-1	SNV-17	MNV-1	MNV-1			DEL-2	DEL-2	DEL-2	DEL-3	INS-1	DEL-2	DEL-3	SNV-1	DEL-2	DEL-1	DEL-2	DEL-4	DEL-4	DEL-1
	DEL-1	INS-1	DEL-1	DEL-1			INS-1										INS-1	INS-1	INS-1	
**VCaP**	DEL-2	DEL-7	SNV-8	SNV-12	SNV-1		SNV-14	SNV-12	SNV-4	SNV-3	SNV-1	SNV-7		INS-1	SNV-2	SNV-1	SNV-3	SNV-3	SNV-3	SNV-4
		SNV-8	MNV-1	MNV-1			DEL-3		DEL-1			DEL-1					INS-1	DEL-2	DEL-1	
		INS-2	DEL-1	DEL-1																
**VCaP-CR**	DEL-2	DEL-7	SNV-7	SNV-12	SNV-1		SNV-14	SNV-12	SNV-4	SNV-3	SNV-1	SNV-7		INS-1	SNV-2	SNV-1	SNV-3	SNV-3	SNV-3	SNV-4
		SNV-8	MNV-1	MNV-1			DEL-3		DEL-1			DEL-1					INS-1	DEL-2	DEL-1	
		INS-2	DEL-1	DEL-1																

## Data Availability

Data used in this publication were generated by projects sponsored by the NCI Physical Sciences in Oncology Initiative.

## Supplementary Materials

The following supporting information can be downloaded at: https://www.mdpi.com/article/10.3390/ijms25116111/s1.
